# Bridging Neurobiology, Heterogeneity, and Comorbidity in Mood Disorders

**DOI:** 10.3390/brainsci15091020

**Published:** 2025-09-22

**Authors:** Balwinder Singh

**Affiliations:** Department of Psychiatry and Psychology, Mayo Clinic, Rochester, MN 55905, USA; singh.balwinder@mayo.edu

Mood disorders are among the most disabling and complex psychiatric conditions, affecting millions worldwide and contributing substantially to morbidity, mortality, and healthcare utilization [1]. Disruptions in the balance between excitatory and inhibitory neurotransmission, particularly within glutamatergic and GABAergic pathways, play a central role in their pathophysiology [2,3,4]. Ketamine has emerged as a rapid-acting antidepressant and is increasingly utilized in treatment-resistant depression [5,6], with intranasal esketamine—the S-enantiomer of ketamine—FDA-approved both as monotherapy and as an adjunct to standard antidepressants. Renewed interest in psychedelics also generates cautious optimism, as preliminary findings suggest potential benefits, though rigorous trials and careful attention to safety, ethics, and long-term outcomes are essential. Meanwhile, novel neuromodulation techniques are reshaping clinical practice by leveraging advances in circuit-based understanding of mood regulation, offering more targeted options for treatment-resistant cases.

This Special Issue, *Clinical Research on Mood Disorders: Opportunities and Challenges*, presents five studies that reflect both the progress and the ongoing challenges in the field. Covering novel therapeutics, disease heterogeneity, epidemiology, and the interface of physical and mental health, these contributions highlight the remarkable opportunities and enduring complexities that continue to shape mood disorder research today.

In a comprehensive systematic review and meta-analysis of (es)ketamine for bipolar depression, Núñez et al. [7] screened 2657 articles, ultimately including 11 in their review and 7 in quantitative synthesis. Despite methodological limitations, including non-randomized studies, both single and repeated ketamine infusions showed comparable short-term efficacy in bipolar depression, with response rates of 54% versus 55%, remission rates of 30% versus 40%, and a relatively modest rate of affective switching. While the evidence base for esketamine remains limited, early findings from small samples appear promising. Together, these results underscore both the therapeutic potential and unanswered questions surrounding ketamine and esketamine as treatments for bipolar depression [8]. In particular, there is a pressing need for well-designed trials to establish long-term efficacy, clarify safety, and develop strategies to minimize treatment-emergent affective switching in bipolar depression [9]. More broadly, this body of work highlights the central importance of glutamatergic and GABAergic modulation as therapeutic targets for mood disorders [2,10].

An important limitation in measuring depression lies in the rating scales themselves, as critical symptoms—such as changes in appetite and eating behavior, which intersect with the well-established relationship between major depression and obesity—are not adequately captured [11,12]. Treviño-Alvarez et al. [13] critically examine the limitations of commonly used depressive symptom rating scales, noting that most fail to assess increased appetite or weight gain—key features in atypical depression and important confounders in studies of antidepressants and obesity. They offer a useful comparison of several depression scales with respect to appetite and weight items, providing a valuable resource for mood disorder researchers. This could inform future studies investigating the relationship between changes in appetite or weight and depression, both at baseline and in response to treatment. Their perspective calls for improved measurement tools that better capture the heterogeneity of depressive presentations, which is essential for both research validity and clinical care.

Anhedonia is a core feature of depression, yet few treatments have demonstrated efficacy in specifically targeting it [14]. Importantly, anhedonia is a risk factor for suicidality in both general and psychiatric populations [14,15]. Prior studies have examined the relationship between the acuity of anhedonia and suicidal thoughts and behaviors [16]. The study by Darquennes et al. [17] investigates the temporal dynamics of anhedonia and its association with suicidal ideation in major depression, revealing that recent changes in anhedonia—rather than lifelong anhedonia—are most strongly linked to suicidal thoughts. These findings underscore the value of nuanced, longitudinal phenotyping for identifying high-risk patients and tailoring interventions.

Depression frequently co-occurs with anxiety, affecting 50–60% of patients with mood disorders [18,19]. A cross-sectional study from South Korea by Kim et al. [20] links low skeletal muscle mass to greater anxiety severity in a large, healthy adult population, revealing notable sex differences: while women reported higher anxiety overall, the association between low muscle mass and anxiety was stronger in men. These findings underscore the importance of integrating somatic health into mood disorder research and point to new avenues for prevention and intervention, while longitudinal studies are needed to clarify causality and implications for quality of life and treatment outcomes.

An important dimension of depression is its interplay with somatic comorbidities, especially cancer. In the United States, breast cancer ranks as the second most common cancer and the second leading cause of cancer-related mortality among women [21]. In an insightful study integrating data from the U.S. National Health and Nutrition Examination Survey with mortality data from the National Death Index, Khubchandani et al. [22] highlighted a higher risk of all-cause mortality among women with both depression and breast cancer compared to those without either condition. They also identified moderating factors for mortality, providing robust epidemiological evidence that depression significantly increases risk among breast cancer survivors. This analysis underscores the importance of integrated care models that address both physical and psychological health, emphasizing the need for collaborative, multidisciplinary approaches in managing comorbid mood and somatic disorders.

Together, the studies in this Special Issue showcase the progress and ongoing challenges in mood disorder research. They highlight advances in rapid-acting treatments like ketamine, while underscoring the need for standardized assessments, translational approaches, and integration of physical and mental health. Nuanced phenotyping, attention to comorbidities, and consideration of developmental and sex-specific factors are essential for identifying high-risk individuals and optimizing outcomes. Collectively, these findings point toward a future driven by innovation, collaboration, and interdisciplinary strategies to improve the lives of those affected by mood disorders.
